# Lung single-cell RNA profiling reveals response of pulmonary capillary to sepsis-induced acute lung injury

**DOI:** 10.3389/fimmu.2024.1308915

**Published:** 2024-01-29

**Authors:** Ruhao Yang, Ting Zheng, Hongyu Xiang, Menglin Liu, Ke Hu

**Affiliations:** ^1^ Department of Respiratory and Critical Care Medicine, Renmin Hospital of Wuhan University, Wuhan, China; ^2^ Department of Emergency, Renmin Hospital of Wuhan University, Wuhan, China; ^3^ Department of Endocrinology, Zhongnan Hospital of Wuhan University, Wuhan, China; ^4^ Department of Rheumatology and Immunology, Zhongnan Hospital of Wuhan University, Wuhan, China

**Keywords:** alveolar capillary cells, acute lung injury, sepsis, immune regulation, single-cell RNA-seq

## Abstract

**Background:**

Sepsis-induced acute lung injury (ALI) poses a significant threat to human health. Endothelial cells, especially pulmonary capillaries, are the primary barriers against sepsis in the lungs. Therefore, investigating endothelial cell function is essential to understand the pathophysiological processes of sepsis-induced ALI.

**Methods:**

We downloaded single-cell RNA-seq expression data from GEO with accession number GSE207651. The mice underwent cecal ligation and puncture (CLP) surgery, and lung tissue samples were collected at 0, 24, and 48 h. The cells were annotated using the CellMarker database and FindAllMarkers functions. GO enrichment analyses were performed using the Metascape software. Gene set enrichment Analysis (GSEA) and variation Analysis (GSVA) were performed to identify differential signaling pathways. Differential expression genes were collected with the “FindMarkers” function. The R package AUCell was used to score individual cells for pathway activities. The Cellchat package was used to explore intracellular communication.

**Results:**

Granulocytes increased significantly as the duration of endotoxemia increased. However, the number of T cells, NK cells, and B cells declined. Pulmonary capillary cells were grouped into three sub-clusters. Capillary-3 cells were enriched in the sham group, but declined sharply in the CLP.24 group. Capillary-1 cells peaked in the CLP.24 group, while Capillary-2 cells were enriched in the CLP.48 group. Furthermore, we found that Cd74+ Capillary-3 cells mainly participated in immune interactions. Plat+ Capillary-1 and Clec1a+ Capillary-2 are involved in various physiological processes. Regarding cell-cell interactions, Plat+ Capillary-1 plays the most critical role in granulocyte adherence to capillaries during ALI. Cd74+ Capillary cells expressing high levels of major histocompatibility complex (MHC) and mainly interacted with Cd8a+ T cells in the sham group.

**Conclusion:**

Plat+ capillaries are involved in the innate immune response through their interaction with neutrophils via ICAM-1 adhesion during endotoxemia, while Cd74+ capillaries epxressed high level of MHC proteins play a role in adaptive immune response through their interaction with T cells. However, it remains unclear whether the function of Cd74+ capillaries leans towards immunity or tolerance, and further studies are needed to confirm this.

## 1 Introduction

Sepsis is a widespread and severe syndrome that arises from uncontrolled host response to infection, leading to organ dysfunction (1). In 2014, Around 1.75 million sepsis cases were recorded in the United States, and approximately half of them required intensive care unit admission (2). A meta-analysis of 27 studies conducted in seven high-income countries estimated that there were approximately 31.5 million cases of sepsis and 19.4 million cases of severe sepsis annually, with a potential 5.3 million deaths (3). A more comprehensive study examining the global, regional, and national incidence of sepsis and mortality in over 100 million individuals revealed that while the incidence of sepsis had decreased from 1990 to 2017, there were an estimated 48.9 million sepsis patients and 19.7% of deaths in 2017 (4). Given its significant morbidity and mortality rates, sepsis is the third leading cause of hospital deaths (5), imposing a significant burden on human health.

The lungs are highly vulnerable to sepsis, which is the leading cause of acute lung injury (ALI) (6). Approximately half of the patients with sepsis develop ALI (7), with a mortality rate of 30–40% (8). ALI is characterized by edema, hyperemia, and inflammatory cell infiltration. Endothelial cells, particularly pulmonary capillaries, form a monolayer that facilitates air exchange. Disruption of the capillary-alveolar barrier in ALI leads to decreased efficiency of air exchange and an influx of interstitial fluid into the pulmonary alveoli. Additionally, endothelial cells can communicate with immune cells such as leukocytes, which are drawn to inflammation sites through adhesion (9).

Endothelial cells serve as the first barrier against sepsis in lungs. A comprehensive understanding of their function during ALI is crucial for gaining insights into the pathophysiological processes of sepsis-induced ALI and the development of more efficient treatments. Pulmonary capillaries surround the alveoli, forming a respiratory surface for oxygen exchange between the blood and the alveoli. Studies have shown that capillaries can be divided into general capillary cells (gCap) and aerocytes (aCap) (10). aCap cells are large cells that extend in a ramified manner around pores, often spanning multiple alveoli. In comparison, gCap cells have a simpler morphology. They are smaller with fewer pores and have less extensive branching, rarely spanning multiple alveoli. In terms of function, aCap cells specialize in gas exchange, while gCap cells manage vascular tone and function as progenitor cells for repair. This indicates that capillaries are not homogeneous; however, there is limited research on the different types of capillaries in sepsis-induced ALI.

To study the multifunctionality of capillaries and their communication with immune cells, we screened single-cell RNA-seq data obtained from the GEO database associated with a sepsis mouse model and lung tissue analysis. We selected and analyzed the dataset GSE207651 to explore changes in capillary phenotype during sepsis-induced ALI. Mice underwent cecal ligation and puncture (CLP), a common technique used to induce polymicrobial sepsis and septic lung injury. Lung tissue samples were collected 0, 24, and 48 h after surgery. CD45- and CD45+ cells were mixed at a 1:1 ratio for single-cell sequencing.

## 2 Material and methods

### 2.1 scRNA-seq expression

We acquired the single-cell RNA-seq gene expression dataset from GEO under accession number GSE207651. Mice aged 6-8 weeks were divided into three groups: sham, CLP.24, and CLP.48. Each group consisted of three mice. The CLP.24 group and CLP.48 group underwent cecal ligation and puncture (CLP) surgery. The sham group and CLP.24 group were sacrificed after 24 hours, while the CLP.48 group was sacrificed after 48 hours. Lung tissue samples from each group were mixed for single-cell RNA sequencing. Magnetic-activated cell sorting (MACS) was used to achieve a ratio of 1:1 for CD45+ cells to CD45- cells.

### 2.2 Analysis of scRNA-seq data

The single-cell library was prepared following the protocol of the 10×Genomics Chromium Next GEM Single Cell 3′ Reagent Kits v3.1. Processed scRNA-seq data were required in the form of raw unique molecular identifier (UMI) count matrix by Cell Ranger (version 5.0.0). We used the Seurat R toolkit (version 4.1.1) for further analyses. The number of cells in each group required from the original data were 8744 cells in the sham group, 12159 cells in the CLP.24 group and 10631 cells in the CLP.48 group. Cells containing fewer than 300 genes or more than 7,500 genes were excluded. Genes expressed in fewer than five cells and those with mitochondrial UMI counts exceeding 10% were excluded. The QC metrics plots can be found in the article/**Supplementary Material**. A total of 29,723 cells remained after filtering for further analysis including 7984 cells in the sham group, 11589 cells in the CLP.24 group and 10150 cells in the CLP.48 group. The data were normalized using Seurat’s NormalizeData function. We selected 2,000 variable genes using the FindVariableGenes function and then applied scaling with the ScaleData function, including the regression of the cell cycle. Principal component analysis based on the variable genes was performed using the RunPCA function. Batch effects were mitigated using the RunHarmony function. The cells were clustered into 36 clusters using FindNeighbors and FindClusters functions with a resolution parameter of 1.2. To re-cluster the endothelial cells (3,801 cells), we used the FindClusters function with a resolution of 0.25, resulting in seven clusters. We employed UMAP to visualize the cluster results.

### 2.3 Marker genes identification and cell type annotation

We employed a double-checking strategy to identify the marker genes. Initially, we identified data-derived marker genes using the FindAllMarkers function. Subsequently, we compared these data-derived marker genes to those reported in the CellMarker database. Additionally, marker genes derived from other studies have been identified (11, 12). Finally, we annotated the cell clusters by manually inspecting the cell types based on both data- and study-derived marker genes. Consequently, we categorized the cells into 7 immune cells and 4 stromal cells. Immune cells were categorized using canonical marker genes, including Cd19, Cd79a, and Cd79b for B cells (n=263 cells); Trbc2 for T cells (n=1317 cells); Gzma for NK cells (n=797 cells); Flt3 and Ccl17 for DC cells (n=516 cells); Mrc1, Marco, and Cd44 for Macrophages (n=1550 cells); Ccr2, F13a1, and Plac8 for Monocytes (n=3514 cells); Retnlg and S100a8 for Granulocytes (n=5846 cells). Epithelial cells (n=796 cells) were identified by Sftpa1, Ager, and Cbr2. Smooth muscle cells (2381 cells) and Fibroblasts (8942 cells) were identified by Tagln, Myh11, Acta2, and Dcn, Gsn, Col1a2, respectively. Endothelial cells (n=3801 cells) were distinguished by the expression of Cdh5, Cldn5, Lyve1, and Calcr1, and were further sub-clustered. Arteries (200 cells), veins (243 cells), and lymphatics (315 cells) clusters were annotated using marker genes (Mgp2 for arteries; Slc6a2 and Bst1 for veins; Fxyd6 and Mmrn1 for lymphatics). Capillaries (2233 cells) were annotated using the marker genes Car4, Lpl, and Fibin, and divided into three types of capillaries (Capillary-1, 939 cells; Capillary-2, 686 cells; Capillary-3, 608 cells).

### 2.4 Go enrichment

GO enrichment analyses were conducted using Metascape, specifically focusing on “biological process” annotations (http://metascape.org/; v3.5; updated date: 2020-09-16). We used “M. musculus” as the species, and the results were filtered using a p-value cutoff of <0.01 (13). The results were visualized using ggplot2 (version 3.3.6).

### 2.5 Gene set variation analysis

Initially, we downloaded the MSigDB Gene Sets for “Mus musculus,” specifying “curated gene sets” and “KEGG” pathways using the “msigdbr” function. Subsequently, we performed a gene set variation analysis using the GSVA package (version 1.42.0).

### 2.6 Gene set enrichment analysis

We conducted differential expression analysis using the “FindMarkers” function. Additionally, we downloaded “c2.cp.kegg.v7.5.1. symbols.gmt” which used as the database. Finally, we performed GSEA analysis by the GSEA function in the clusterProfiler package (version 4.2.2). The results were presented using the gseaplot2 function in the enrichplot package (version 1.14.2) or interpreted based on the normalized enrichment score (NES) and adjusted p-value.

### 2.7 Differential expression analysis

Differentially expressed genes were identified using the “FindMarkers” function, applying criteria of “|avg_log2FC| > 1.2” and “p_val_adj < 0.05” to define the significant DEGs.

### 2.8 Pathway activity

We utilized the R package AUCell to assess the pathway activities in individual cells. Initially, we computed gene expression rankings for individual cell using “AUCell_buildRankings” function. The canonical KEGG pathway database “c2.cp.kegg.v7.5.1. symbols.gmt” was obtained from the Broad Institute’s website. Area-under-the-curve (AUC) values were determined by utilizing the “AUCell_calcAUC” function, which relies on gene expression rankings.

### 2.9 Interactions between cells

The Cellchat package was used to investigate intracellular communication within all endothelial cell types and between T cells or granulocytes and endothelial cell subpopulations. We utilized the mouse database, which includes categories such as “Secreted Signaling,” “Cell-Cell Contact,” and “ECM-Receptor” from CellchatDB. Communication probabilities were analyzed across the three conditions.

## 3 Results

### 3.1 Single-cell RNA-seq validates pulmonary cell composition and changes during endotoxemia

**Figure 1:**
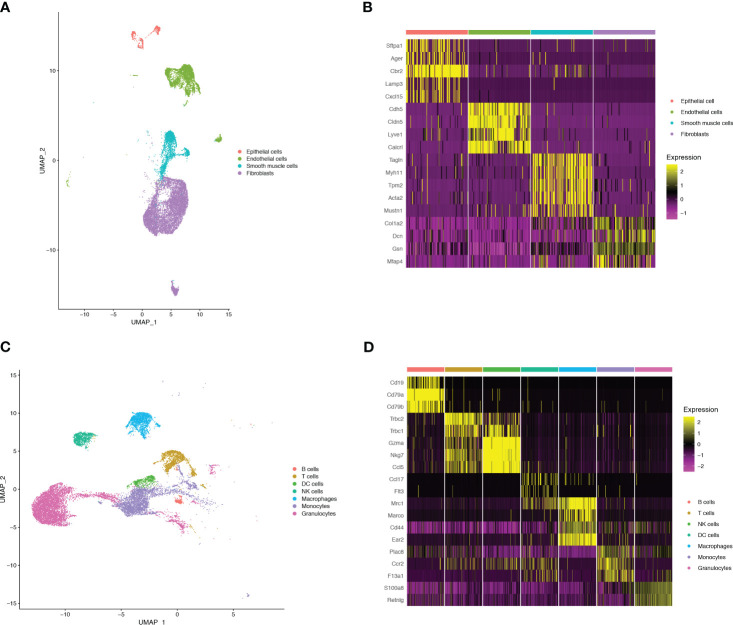
Single-cell RNA-seq identifies pulmonary cellular composition in endotoxemia. **(A)** UMAP plot of 4 non-immune cells clusters in mouse lungs. **(B)** Heatmap showing log-transformed expression of marker genes for each non-immune cluster. **(C)** UMAP plot of 7 immune cells clusters in mouse lungs. **(D)** Heatmap showing log-transformed expression of marker genes for each immune cluster.

**Figure 2:**
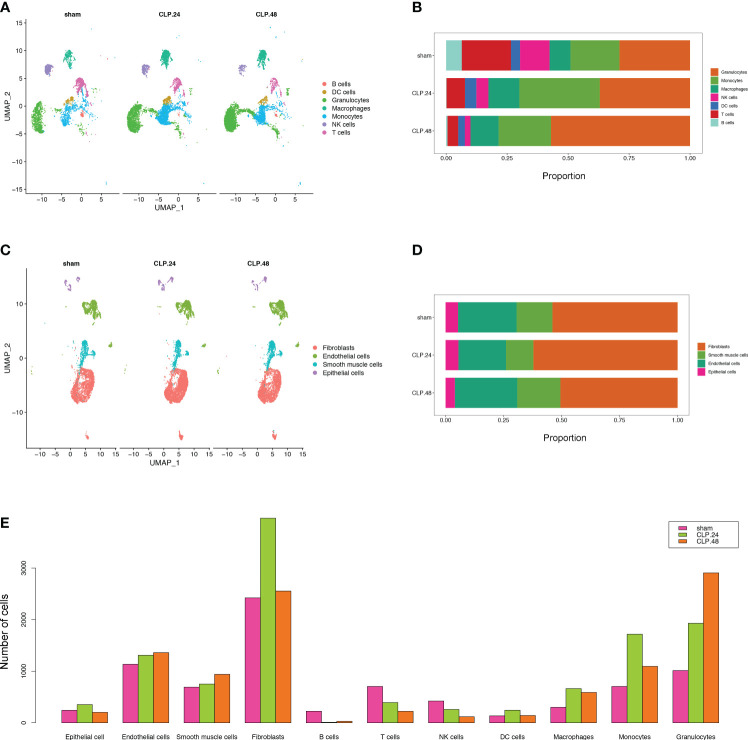
Single-cell RNA-seq identifies pulmonary cellular alterations in endotoxemia. **(A)** UMAP plot of 7 immune cells clusters in different group. **(B)** Proportional graph displaying the proportion of each immune cell type in different groups. **(C)** UMAP plot of 4 non-immune cells clusters in different group. **(D)** Proportional graph displaying the proportion of each non-immune cell type in different groups. **(E)** Bar graph showing the number of each cell type in different groups.

To monitor alterations in pulmonary cell composition during endotoxemia, we conducted scRNA-seq analysis of CD45+ and CD45- cells extracted from mouse lung tissue at three time points. We defined four cell types in CD45- cells (15920 cells) (**Figure 1A**): Fibroblasts (Col1a2, Dcn, Gsn, Mfap4), Endothelial cells (Cdh5, Cldn5, Lyve1, Calcrl), Smooth muscle cells (Tagln, Myh11, Tpm2, Acta2), and Epithelial cells (Sftpa1, Ager, Cbr2, Lamp3, Cxcl15) (**Figure 1B**). We identified seven cell types among CD45+ cells (13803 cells) (**Figure 1C**): B cells (Cd19, Cd79a, Cd79b), DC cells (Ccl17,Flt3), Granulocytes (S100a8, Retnlg), Macrophages (Mrc1, Marco, Cd44, Ear2), Monocytes (Plac8, Ccr2, F13a1), NK cells (Gzma, Nkg7, Ccl5), and T cells (Trbc2, Trbc1) (**Figure 1D**). Compared with those in the sham group, there were significant alterations in CD45+ cells. Granulocytes expanded, whereas B cells, T cells, and NK cells shrank in an endotoxemia time-dependent manner. Macrophages and monocytes increased in the CLP.24 group, but remained in the CLP.48 group (**Figures 2A, B**). Among CD45- cells, the number of epithelial cells decreased slightly in an endotoxemia time-dependent manner. The number of endothelial cells and smooth Muscle cells decreased at 24 h in the CLP group but showed a rebound at 48 h (**Figures 2C–E**).

### 3.2 Alterations in Endothelial cell subtypes during endotoxemia

**Figure 3:**
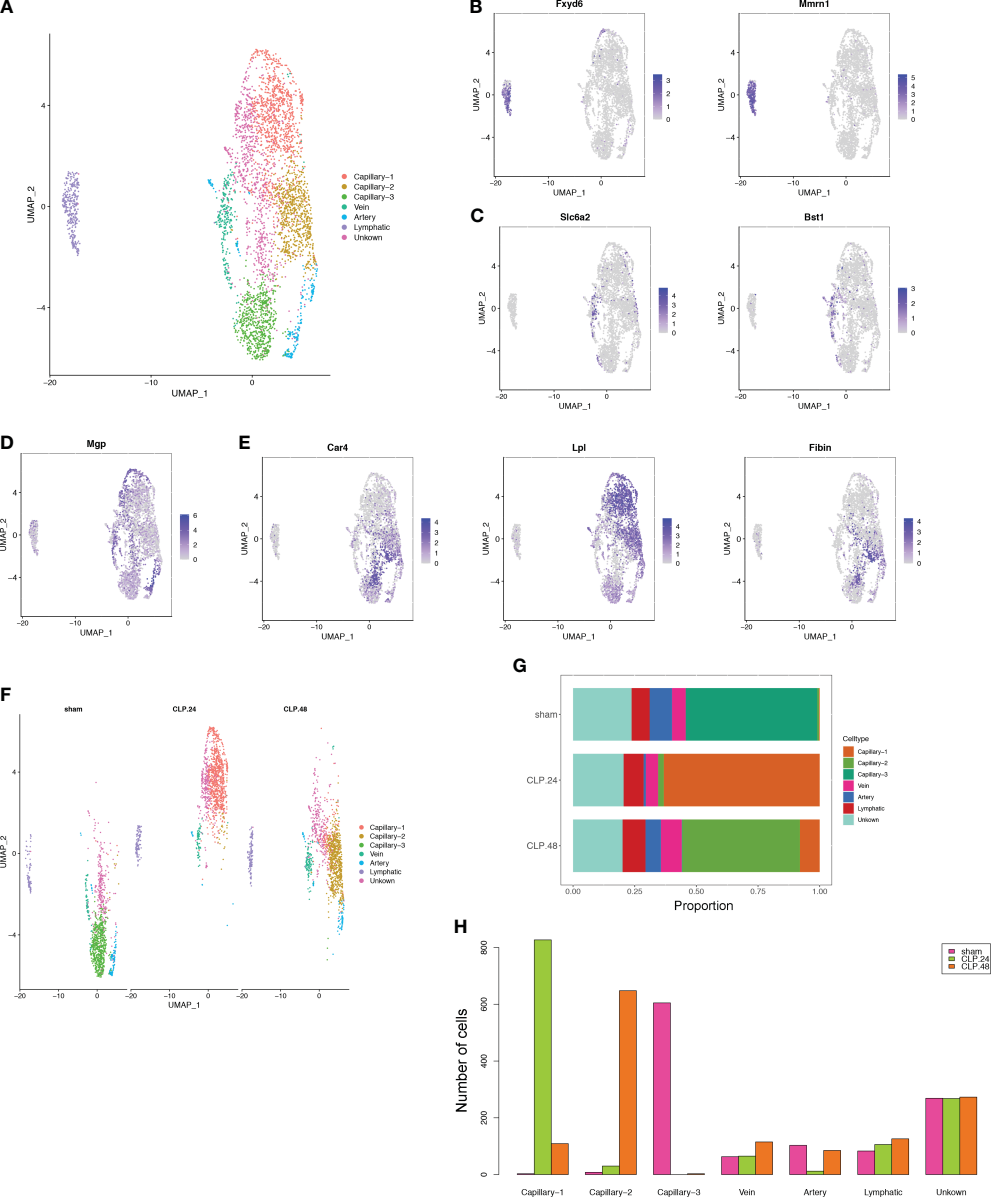
Alterations in endothelial cell transcriptional profiles in endotoxemia. **(A)** UMAP plots depicting 7 endothelial cell clusters in the lungs of mice. **(B–E)** Feature plots illustrating the expression scores of marker genes for lymphatic cells (Fxyd6, Mmrn1), vein cells (Scl6a2, Bst1), artery marker genes (Mgp), and capillary cells (Car4, Lpl, Fibin) across various cell clusters. **(F)** UMAP plot of 7 endothelial cell clusters in different groups. **(G)** Proportional graph displaying the proportions of each endothelial cell type in the different groups. **(H)** Bar graph showing the number of each endothelial cell type in different groups.

Given the critical role of endothelial cells in ALI. We further subdivided endothelial cells into seven clusters (**Figure 3A**). Three clusters among them were annotated as lymphatic (Fxyd6, Mmrn1) cells (**Figure 3B**), vein (Slc6a2, Bst1) (**Figure 3C**) and artery (Mgp) (**Figure 3D**). There were no significant marker genes in one cluster; therefore, we identified this as an unknown cell type. The other three clusters showed capillary marker genes (Car4, Lpl, and Fibin); therefore, we identified them as Capillary-1, Capillary-2, and Capillary-3 (**Figure 3E**). The proportions of the three capillary clusters exhibited significant changes during endotoxemia (**Figure 3F**). In the sham group, Capillary-3 was the main cell type, whereas in the CLP group at 24 h, Capillary-1 was the main cell type and Capillary-3 decreased to zero. Capillary-2 expanded in an endotoxemia time-dependent manner, peaking at 48 h (**Figures 3G, H**). The change in the total number of endothelial cells between groups was not obvious, while the alteration of capillary subsets was evident. This indicates that the change in capillary subsets is crucial during endotoxemia.

### 3.3 Pulmonary capillary subtype phenotype and function

**Figure 4:**
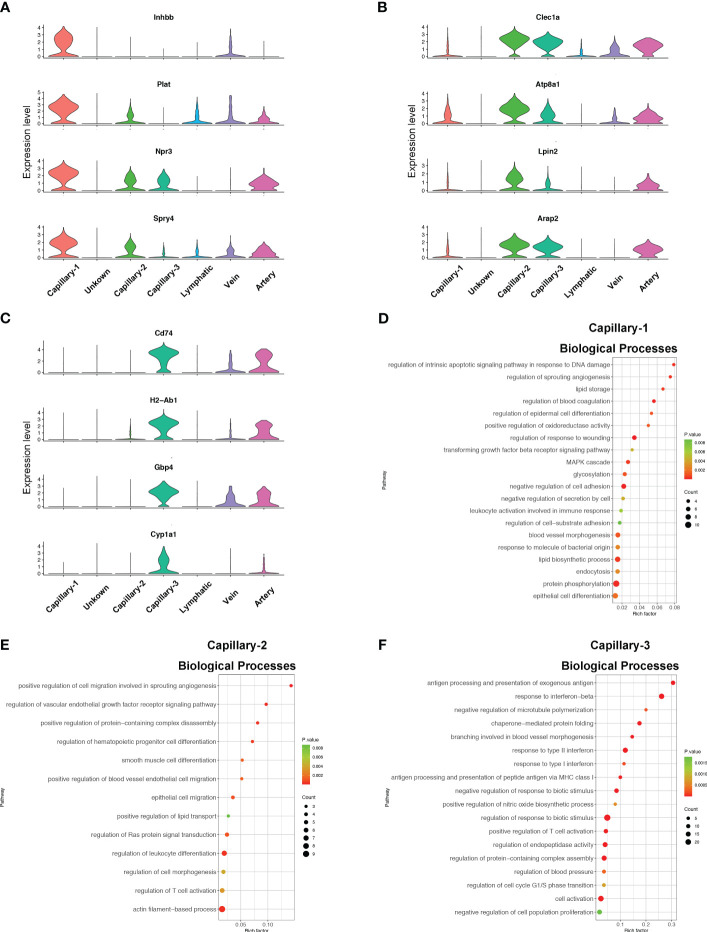
Phenotype and function of pulmonary capillary subtypes in sepsis. **(A–C)** Violin plots displaying the normalized log-transformed expression levels of representative genes used to define Capillary-1 (Inhbb, Plat, Npr3, Spry4), Capillary-2 (Clec1a, Atp8a1, Lpin2, Arap2) and Capillary-3 (Cd74, H2-Ab1, Gbp4, Cyp1a1) respectively. **(D–F)** Dot plots illustrating the major signaling pathways terms of marker genes in three capillary subtypes through GO analysis (Biological processes).

**Figure 5:**
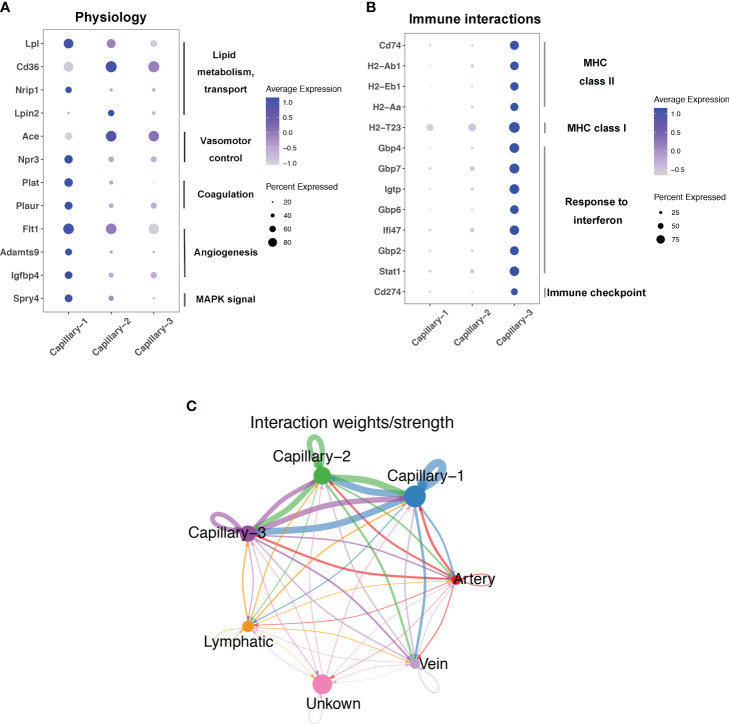
Function and interaction of Pulmonary capillary subtype in sepsis. **(A, B)** Dot plots showing expression in each capillary subtype for selected differentially expressed genes associated with physiology process or immune interactions. **(C)** Circle plots showing interaction weights of ligand-receptor in different endothelial cell type.

Endothelial cell subtypes showed significant changes in capillary phenotypes, highlighting the crucial role of pulmonary capillaries in endotoxemia. We identified marker genes for the three different capillary clusters in endothelial cells. Inhbb, Plat, Npr3, and Spry4 were highly expressed in Capillary-1 cells (**Figure 4A**). Clec1a, Atp8a1, Lpin2, and Arap2 were highly expressed in Capillary-2 (**Figure 4B**). Capillary-3 cells expressing high levels of Cd74, H2-Ab1, Gbp4 and Cyp1a1 (**Figure 4C**). Biological processes were examined to investigate capillary subtype function. Regulation of sprouting angiogenesis, lipid storage, regulation of blood coagulation, and the Microtubule-Associated Protein Kinase (MAPK) cascade were enriched in Capillary-1 cells (**Figure 4D**). Capillary-2 showed vascular endothelial growth factor receptor signaling and lipid transport enrichment (**Figure 4E**). Antigen processing and presentation, and response to interferon were enriched in Capillary-3 (**Figure 4F**). Moreover, we explored highly expressed genes and their functions in each capillary subset. We found that Plat, Plaur, Lpl and Flt1 were enriched in capillary-1 cells, while Flt1 and CD36 were enriched in capillary-2 cells. Among them, Plat and Plaur are related to blood coagulation (14). Flt1 encodes the receptor for the pro-angiogenic family and plays important roles in angiogenesis (15). Lpl encodes lipoprotein lipase which is a crucial enzyme in lipid metabolism (16). CD36, as an adipocyte receptor, optimizes tissue fatty acid uptake in endothelial cells (17). Capillary-3 was involved in adaptive immune response with high expression of Cd74, Stat, Cd274 (**Figures 5A, B**). Interactions within endothelial cells suggested stronger interactions among capillary subtypes (**Figure 5C**).

### 3.4 Alteration of signaling pathway during endotoxemia in endothelial cells

**Figure 6:**
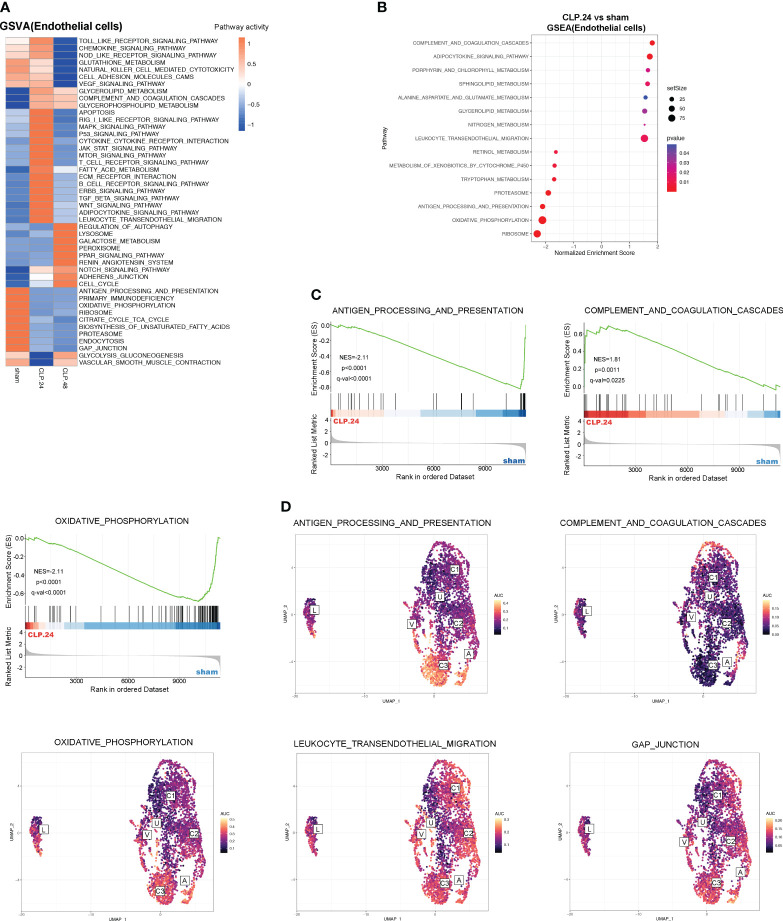
GSVA and Differential KEGG signaling pathway in endothelial cells between sham and CLP.24 group. **(A)** Heatmap showing GSVA analysis of KEGG signaling pathway in endothelial cells from each group. **(B)** Dot plots illustrating GSEA analysis of KEGG signaling pathway in endothelial cells between sham group and CLP.24 group. **(C)** GSEA analysis of selected KEGG signaling pathway comparing sham group and CLP.24 group. **(D)** Distribution of selected KEGG signaling pathway activity AUC scores across all samples.

**Figure 7:**
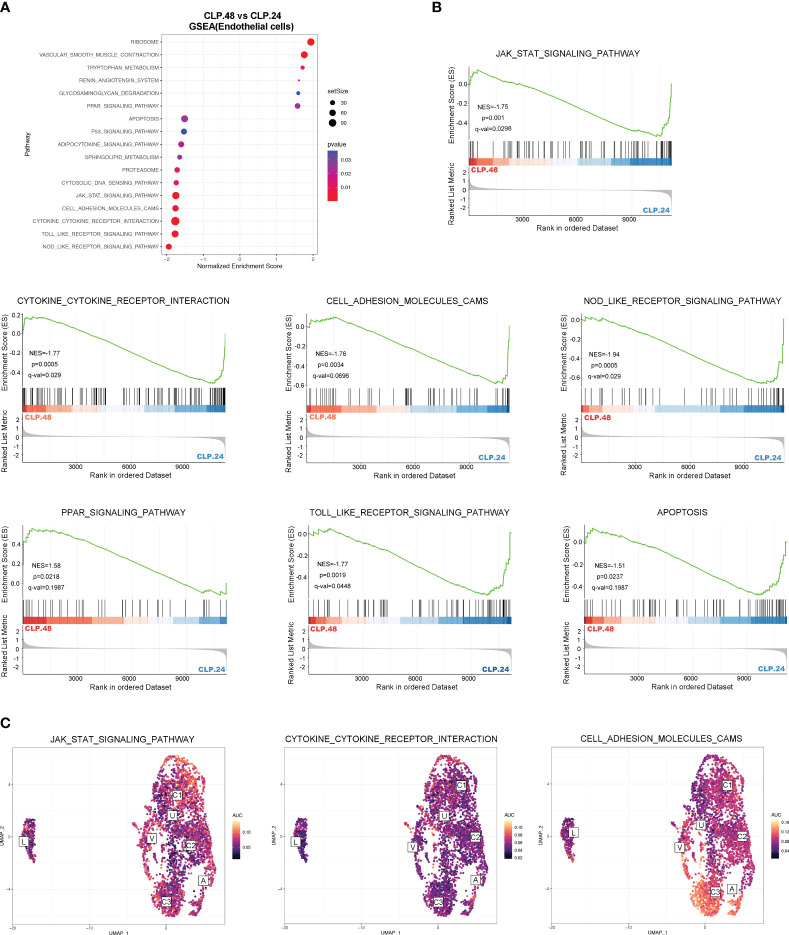
Differential KEGG signaling pathway in endothelial cells between CLP.24 and CLP.48 group. **(A)** Dot plots illustrating GSEA analysis of KEGG signaling pathway in endothelial cells between the CLP.24 group and CLP.48 group. **(B)** GSEA analysis of selected KEGG signaling pathway comparing the CLP.24 group and CLP.48 group. **(C)** Distribution of selected KEGG signaling pathway activity AUC scores across all samples.

To explore the alterations in signaling pathways during endotoxemia in endothelial cells, we performed GSEA and GSVA (**Figure 6A**). The results showed that antigen processing and presentation, and oxidative phosphorylation were decreased in the CLP group at 24 h compared to those in the sham group, whereas complement and coagulation cascades, and leukocyte transendothelial migration were increased (**Figures 6B, C**). The AUCell results indicated that Capillary-3 primarily played a role in antigen processing and presentation, as well as oxidative phosphorylation, while Capillary-1 was associated with complement and coagulation cascades (**Figure 6D**). Compared to the CLP group at 24 h, Jak-Stat, cytokine receptor interaction, Nod-like receptors, cell adhesion molecules, and Toll-like receptor signaling pathways were alleviated in the CLP group at 48 h, while the peroxisome proliferator activated receptor (PPAR) signaling pathway was stronger (**Figures 7A, B**). The AUcell results showed that the Jak-Stat signaling pathway and cytokine receptor interactions were enriched in Capillary-1 cells (**Figure 7C**).

### 3.5 Alteration of gene during endotoxemia in endothelial cells

**Figure 8:**
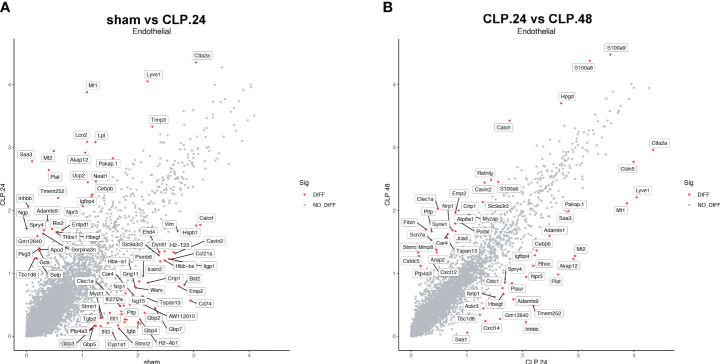
Differential gene expression during endotoxemia in endothelial cells. **(A)** Scatter plots displaying median normalized log-transformed expression levels with log1p() function in endothelial cells comparing sham group and CLP.24 group. **(B)** Scatter plots displaying median normalized log-transformed expression levels with log1p() function in endothelial cells comparing CLP.24 group and CLP.48 group.

Differential genes were identified using the FindMarkers function. The expression of genes, including Plat, Lpl, Neat1, Npr3, Igfbp4, Entpd1, Apod, Thbs1, and Selp, was upregulated in the CLP group at 24 h compared to that in the sham group (**Figure 8A**). Among these, the protein encoded by Plat can lead to hyperfibrinolysis. Lipoprotein lipase encoded by Lpl is an essential enzyme in lipid metabolism that can increase the production of fatty acids. Neat1 is a long noncoding RNA that is considered an oncogene. Neat1 aggravates sepsis-induced lung injury. Igfbp4, Entpd1, and Apod regulate angiogenesis. Thbs1 is an adhesive glycoprotein that mediates cell-cell or cell-matrix interactions. Selp encodes selectin P, which mediates endothelial-leukocyte interactions. The expression of genes related to antigen presentation, including Gbp4, Gbp7, Cd74, H2-Ab1, H2-Eb1, and Stat1, significantly decreased in the CLP group at 24 h. S100a8, Jcad, Cxcl12, S100a6, Cavin2, Pltp, Nrp1, and Ptp4a3 were upregulated in the CLP group at 48 h compared with those in the CLP group at 24 h (**Figure 8B**). Some genes were differentially expressed in the CLP.24 group compared to the sham group but showed a reverse pattern in the CLP.48 group, including Plat, Spry4, Npr3, Igfbp4, Cavin2, Pltp, Nrp1, and Ptp4a3.

### 3.6 The communication between endothelial and granulocytes

**Figure 9:**
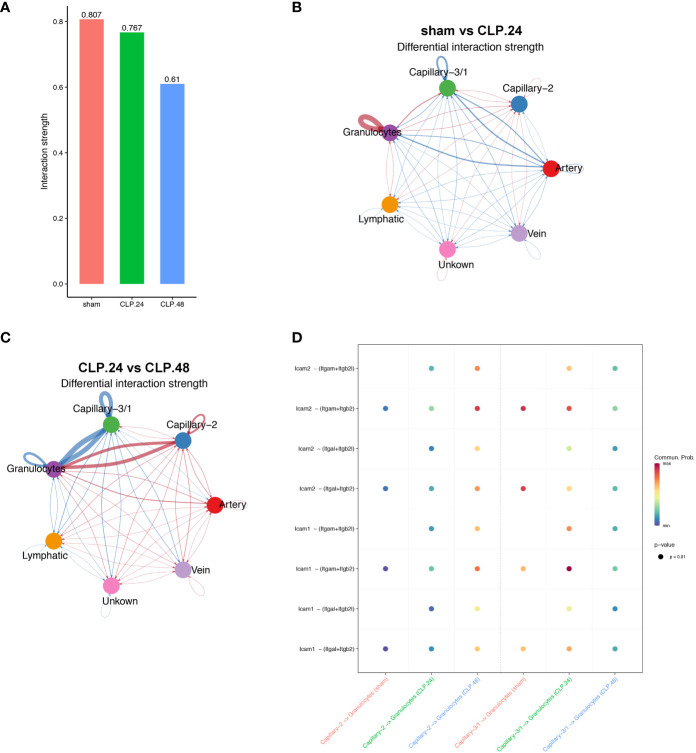
Communication between endothelial and granulocytes. **(A)** Bar plot showing interaction strength between granulocytes and endothelial cells in different group. Capillary-3/1 represented capillary-3 in the sham group and capillary-1 in the CLP.24 group. **(B)** Circle plots displaying differential interaction weights comparing the sham group and CLP.24 group in different endothelial cell types. **(C)** Circle plots showing differential interaction weights comparing of CLP.24 group and CLP.48 group in different endothelial cell types. **(D)** Dot plots illustrating ligand-receptor communication of ICAM signaling pathway between each capillary cell type and granulocytes in different group.

Since the alteration of endothelial cells during endotoxemia will result in a change in communication between endothelial cells and other cell types, furthermore, immune cells are the most involved in endotoxemia. Therefore, we investigated the communication between endothelial cells and certain immune cells. Neutrophils serve as the initial defense during acute inflammation and enter the circulation in large numbers. Their migration from circulation into lung tissues involves a trans-endothelial process. GSEA results showed that leukocyte transendothelial migration was elevated in the CLP group at 24 h compared to that in the sham group. Cellchat was used to examine communication between endothelial cells and neutrophils (**Figure 9A**). In the sham group, capillary-1 was excluded because it contained only three cells. In the CLP.24 group, capillary-3 was excluded because it contained no cells. The results revealed that communication among granulocytes was strengthened in the CLP.24 group, whereas the changes in communication between granulocytes and endothelial cells were not significant (**Figure 9B**). In the CLP.48 group, communication between granulocytes and capillary-2 increased significantly, whereas communication between granulocytes and capillary-1 decreased significantly (**Figure 9C**). Ligand-receptor analysis indicated that intercellular cell adhesion molecule-1 (ICAM-1) found in endothelial cells interacted with granulocytes, with a heightened interaction observed in the CLP group. In the CLP.24 group, capillary-1 primarily participated in the interaction, whereas capillary-2 played a more prominent role in this interaction in the CLP.48 group. In contrast to ICAM-1, intercellular cell adhesion molecule-2 (ICAM-2) was decreased in the CLP.24 group compared to that in the sham group. As a ligand, the strength of the interaction between granulocytes and endothelial cells changed depending on the receptor (**Figure 9D**).

### 3.7 The communication between endothelial and T cells

**Figure 10:**
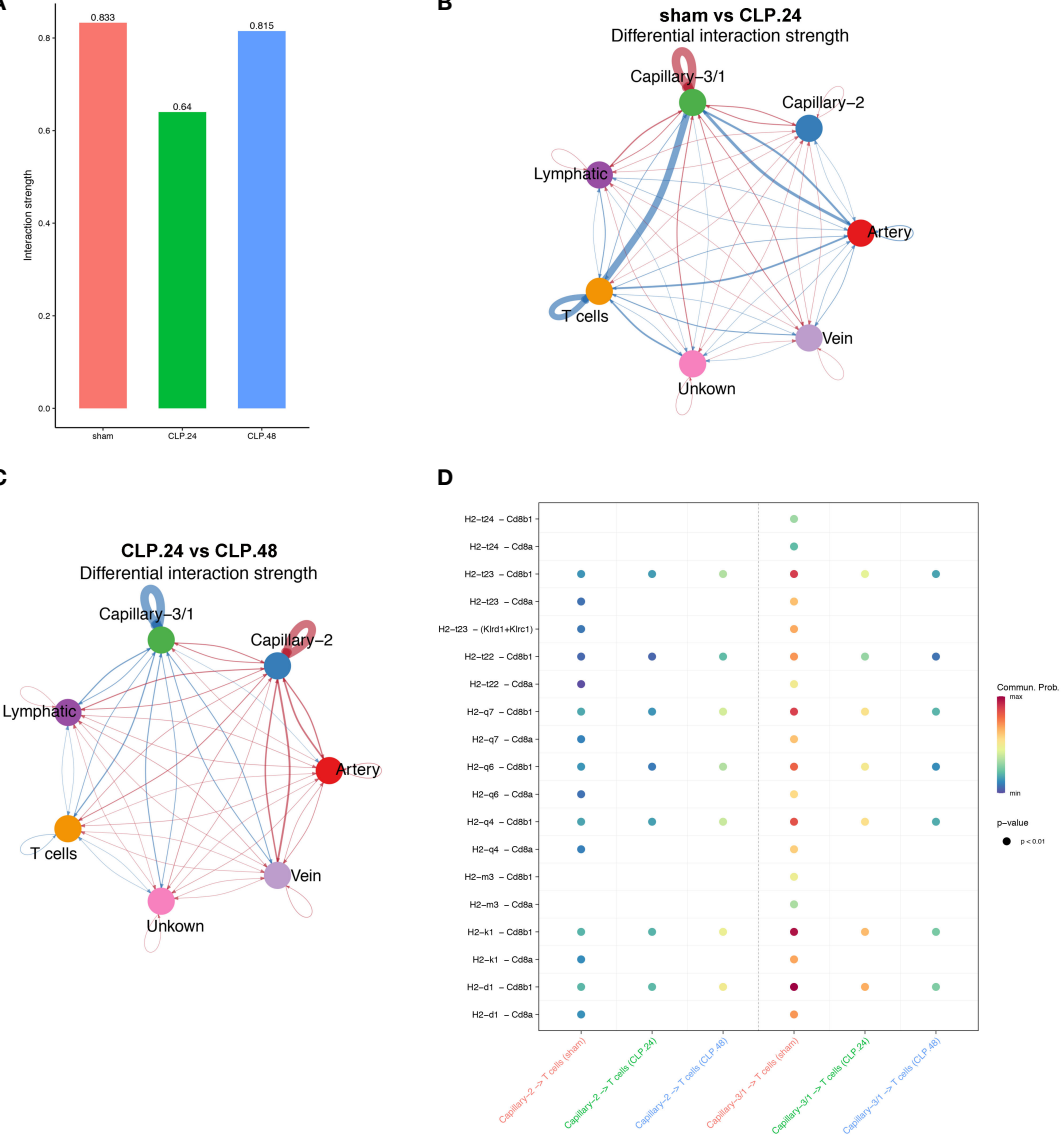
Communication between endothelial and T cells. **(A)** Bar plot showing interaction strength between T cells and endothelial cells in different groups. Capillary-3/1 represents capillary-3 in sham group and capillary-1 in CLP.24 group. **(B)** Circle plots showing differential interaction weights comparing sham group and CLP.24 group in different endothelial cell types. **(C)** Circle plots displaying differential interaction weights comparing CLP.24 group and CLP.48 group in different endothelial cell types. **(D)** Dot plots illustrating ligand-receptor communication of the MHC signaling pathway between each capillary cell and T cells in different groups.

As the antigen processing and presentation signaling pathways changed significantly in the capillaries during endotoxemia, we hypothesized that capillaries might be involved in communication with T cells. The results indicated that the interaction between T cells and capillary-1 was significantly weaker than that between T cells and capillary-3. As the ratio of capillary-3 to capillary-1 decreased, the interaction strength also decreased in the CLP.24 group in comparison to that in the sham group (**Figures 10A–C**). Ligand-receptor analysis also suggested that capillary-T cell communication was noticeably weakened when CD8 acted as a receptor during endotoxemia, indicating a decrease in the MHC-I signaling pathway. There were no notable alterations in the interaction between CLP groups at 24 and 48 h (**Figure 10D**).

## 4 Discussion

**Figure 11:**
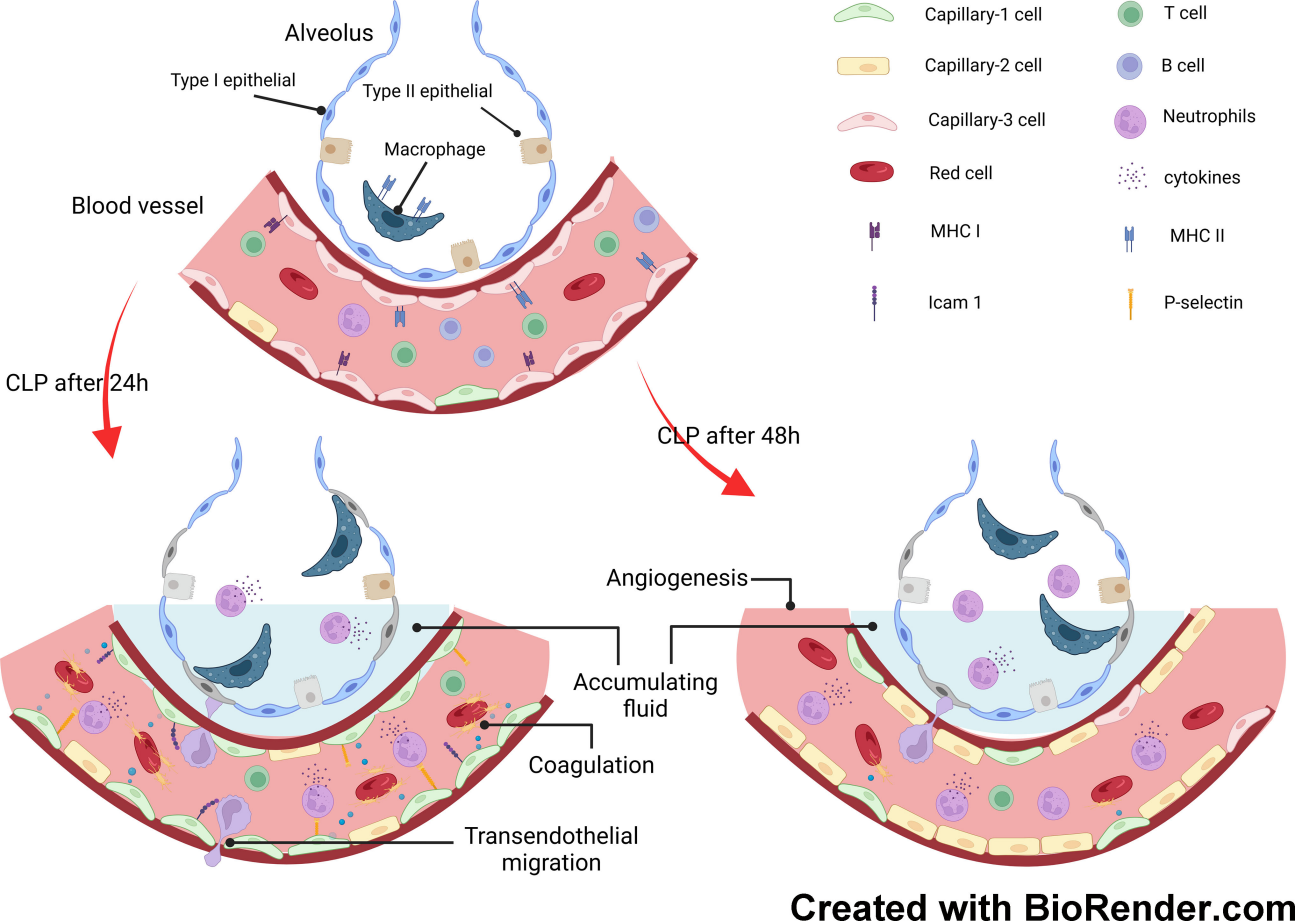
Mechanisms underlying response of pulmonary capillary to sepsis-induced acute lung injury.

In this study, we explored the transcriptomics of pulmonary cells and their genotypic features in a mouse model of endotoxemia. Granulocyte levels increased significantly as the duration of endotoxemia increased. However, the number of NK, T, and B cells decreased. To investigate the involvement of pulmonary capillary cells in sepsis-induced ALI, we categorized them into three subclusters. Capillary-3 cells, marked by elevated Cd74 gene expression, were enriched in the sham group, but experienced a significant decline in the CLP.24 group. Capillary-1 cells, with high expression of the Plat gene, peaked in the CLP.24 group, whereas Capillary-2 cells, with high expression of the Clec1a gene, were enriched in the CLP.48 group. Furthermore, we observed that Cd74+ capillary cells primarily participated in immune interactions related to antigen processing and presentation. Plat+ capillary-1 and Clec1a+ capillary-2 are involved in physiological processes such as coagulation, vasomotor control, lipid metabolism and transport. Regarding cell-cell interactions, Plat+ capillary-1 played the most significant role in granulocyte adherence to capillaries during ALI. In the sham group, Cd74+ capillary cells expressing high levels of major histocompatibility complex (MHC) proteins and mainly interacting with Cd8a+ T cells (**Figure 11**).

The alteration of signaling pathways in endothelial cells during endotoxemia mainly includes the immune response, coagulation cascades and metabolism. The alteration of the immune response was the most evident. Antigen processing and presentation were inhibited in the CLP group, while the inflammatory signal pathways including Jak-Stat, leukocyte transendothelial migration, cytokine receptor interaction, Nod-like receptors, and Toll-like receptor signaling pathways peaked in the CLP.24 group, but were significantly suppressed in the CLP.48 group. As for coagulation cascades, they remained at a high level in the CLP group. Metabolism alteration was also observed in endothelial cells, especially oxidative phosphorylation which was mostly suppressed during endotoxemia.

Neutrophils function as an initial defense against invading microorganisms and are components of the innate immune system. Under healthy conditions, neutrophils are mostly retained in the bone marrow and remain in circulation in small numbers (18). However, under inflammatory conditions, a large number of neutrophils are released into circulation and accumulate in the endothelium at the site of infection or damage. Our results showed a significant increase in the number of neutrophils in the ALI (19). Neutrophil tethering and adhesion to the endothelial surface are the first steps of transendothelial migration. P-selectin on the endothelium mediates the tethering and rolling of neutrophils in injured vascular beds (20). Our differential gene expression results showed a significant increase in Selp (encoding P-selectin) expression at 24 h during endotoxemia. Intercellular cell adhesion molecules (ICAMs) are transmembrane glycoproteins of the immunoglobulin superfamily, which are essential for neutrophil adhesion and trafficking into tissues. ICAM-1, initially expressed at modest levels on the endothelial surface, is upregulated via pro-inflammatory signaling pathways (21). Numerous studies have demonstrated that ICAM-1 plays a central role in facilitating firm leukocyte adhesion and in supporting neutrophil extravasation by increasing endothelial cell stiffness (22, 23) (24),. In contrast, ICAM-2 was highly expressed in the endothelium. Amsellem et al. demonstrated that vascular permeability increases in ICAM-2-deficient mice (25), indicating that ICAM-2 regulates the maturation of endothelial junctions (26). Our study showed that ICAM-1 levels increased at 24 h and declined at 48 h during endotoxemia. ICAM-1 expressed in capillary-1 cells has stronger interaction with neutrophil than ICAM-1 expressed in capillary-2 cells, which mediates neutrophil adhesion during sepsis. ICAM-2, on the other hand, decreased during endotoxemia in our study, which may have contributed to increased vascular permeability; however, the change in the interaction between ICAM-2 and neutrophils for adhesion was not obvious.

In addition to hyperinflammation, sepsis is also characterized by immunosuppression. Immunosuppression is characterized by damage to both innate and adaptive immune systems, including a reduction in T cells (27, 28), impaired lymphocyte response (29), and damaged phagocyte functions (30). In contrast to neutrophils, we found that T cells in the lungs decreased during endotoxemia. T cells participate in adaptive immunity and are essential for the maintenance of immune homeostasis. A previous study has shown that T cell depletion is associated with increased mortality (31). Additionally, Sun et al. found that T cell levels in the liver decreased during lipopolysaccharide (LPS)-induced sepsis, and part of this T cell suppression was attributed to the interaction between PD-1 and PD-L1 in endotoxemia (32). We also observed a reduction in T cells within the lung during endotoxemia, which is regarded as immunosuppression (33).

Endothelial cells are increasingly acknowledged as participants in the immune response to injuries, in addition to their roles in regulating vascular barrier function, modulating vasomotor tone, and controlling coagulation and hemostasis (34, 35). We investigated the heterogeneity of pulmonary capillaries and their function in sepsis-induced ALI. The capillary cells were clustered into three subtypes. Plat+capillary-1, with high expression of Plat and Plaur, indicated its anticoagulant and procoagulant properties, which were also verified by biological process enrichment in GO analysis. Plat+capillary-1 levels increased significantly after endotoxemia stimulation, indicating an association between Plat+ capillary-1 levels and the coagulation process in ALI. Both Plat+ capillary-1 and Clec1a+ capillary-2 participated in vasomotor control, angiogenesis, lipid metabolism, and transport, whereas Cd74+ capillary-3 contributed to the immune response in ALI. Endothelial cells are not classically viewed as immune cells; however, studies have shown that they express innate immune receptors, including toll-like receptors (TLRs) (36), NOD-like receptors (37) and RIG-I like receptors (38). Our results also showed that the signaling pathways peaked in the CLP.24 group and declined in CLP.48 group in endothelial cells (**Figure 6A**). Moreover, endothelial cells are also involved in the adaptive immune response. Endothelial cells do not serve as dedicated antigen-presenting cells (APCs); however, under specific conditions, such as inflammatory responses, they are capable of expressing MHC class II molecules and function as antigen-presenting cells (APCs). Previous studies have shown that capillary cells express MHC class II molecules in the heart (39), kidney (40) and liver (41) during transplantation. However, studies of MHC molecules in pulmonary endothelial cells are lacking. In our study, capillary-3 highly expressing genes associated with MHC proteins, including Cd74, H2-Ab1, H2-Eb1, H2-Aa, and H2-T23, indicating that Cd74+capillary-3 possibly participated in the adaptive immune response. In contrast to the upregulation during inflammation, MHC molecule expression was downregulated in the lungs of mice with sepsis-induced ALI in our study. Some studies have demonstrated that low levels of human leukocyte Antigen DR (HLA-DR) expression are linked to immune dysfunction and decreased survival in patients with sepsis related immunosuppression (42, 43). One possible explanation for the decrease of MHC molecule expression in CLP group may be immunosuppression. However, some studies have reported no correlation has been found between HLA-DR and survival in patients with sepsis (44). Another possible explanation for the change in expression of MHC molecules could be the disturbance of the balance between tolerance and immunity. Alveoli are exposed to non-sterile air, and aerosolized pathogens can be found in the alveoli (45). Unlike the skin, which is dense tissue and covered with a cuticle to prevent invasion by pathogens, the alveoli are thin and vulnerable. Although a strong inflammatory response can eliminate pathogens, it can also cause tissue damage. Neupane et al. discovered that patrolling alveolar macrophages conceal bacteria from the immune system in order to prevent harmful inflammation in the lungs (46). In our study, although the pulmonary capillary, a part of the alveoli, expressed a high level of MHC molecules in the sham group, it did not induce severe inflammation. This could be explained by T cell tolerance after antigen presentation by the pulmonary capillary. In the liver, Limmer and Von et al. found that liver sinusoidal endothelial cells have the ability to cross-present soluble exogenous antigens to CD8+ T cells via MHC-I molecules, resulting in T cell tolerance rather than immunity (47, 48). In our study, the decrease in MHC molecules in the ALI group may suggest that immune tolerance was disrupted, leading to inappropriate inflammation.

The data used in our study was provided by Wang, F et al. (49). The original study focused on alterations of all cell types in the lung during sepsis. Our study primarily analyzed endothelial cells, specifically capillary cells, in the lung during sepsis. We also investigated the subset of pulmonary capillary cells and their function and signaling pathways during sepsis. Additionally, we evaluated the communication between capillary cells and important immune cells involved in sepsis.

There were several limitations in our research. Firstly, the data used in our study was obtained from a public database rather than original data. Secondly, the database we used was not sufficient. The model mice in our study were established through CLP surgery, but it would be more credible if data associated with mice models established by LPS or human samples were included. Thirdly, our study only included experiments conducted *in vivo*. It would be more comprehensive if experiments conducted *in vitro* were included, as this would provide a better understanding of the mechanism.

## 5 Conclusion

In conclusion, we demonstrated changes in various cellular characteristics, particularly in pulmonary capillary cells within the lungs, during sepsis using single-cell sequencing analysis. Different capillary clusters have been shown to participate in different physiological processes. Regarding the immune response, Plat+ capillary-1 are engaged in the innate immune response through interaction with neutrophils via ICAM-1 adhesion during endotoxemia. In contrast, Cd74+ capillaries epxressed high level of MHC proteins play a role in adaptive immune response through their interaction with T cells. However, it remains unclear whether the function of Cd74+ capillaries leans towards immunity or tolerance, and further studies are needed to confirm this.

## Data availability statement

The code used to analyze the data in this study can be found in online repositories. The names of the repository/repositories and accession number(s) can be found in the article/**Supplementary Material**. Further inquiries can be directed to the corresponding author.

## Author contributions

RY: Conceptualization, Writing – original draft. TZ: Formal Analysis, Visualization, Writing – original draft. HX: Methodology, Writing – review & editing. ML: Validation, Writing – review & editing. KH: Writing – review & editing.
