# Supplementary material for: Lung single-cell RNA profiling reveals response of pulmonary capillary to sepsis-induced acute lung injury
Source: Front Immunol. 2024 Jan 29;15:1308915. doi: 10.3389/fimmu.2024.1308915 (PMC10859485; doi:10.3389/fimmu.2024.1308915)
Supplement: Supplementary file 1 [file DataSheet_1.pdf]

```
Sys.setenv(LANGUAGE = "en")
options(stringsAsFactors = FALSE)
rm(list=ls())

setwd("F:/ GSE207651_RAW")
library(Seurat)
library(tidyverse)
library(dplyr)
library(patchwork)
library(devtools)
library(harmony)

scRNA.sham=Read10X("sham/")
scRNA.CLP.24=Read10X("CLP/")
scRNA.CLP.48=Read10X("CLP-48/")
scRNA.sham = CreateSeuratObject(scRNA.sham, min.cells = 5, min.features = 300)
scRNA.CLP.24=CreateSeuratObject(scRNA.CLP.24, min.cells = 5, min.features = 300)
scRNA.CLP.48=CreateSeuratObject(scRNA.CLP.48, min.cells = 5, min.features = 300)

####################################################
scRNA.sham[["percent.mt"]] <- PercentageFeatureSet(scRNA.sham, pattern = "^mt-")
col.num <- length(levels(scRNA.sham@active.ident))
violin <- VlnPlot(scRNA.sham,
                  features = c("nFeature_RNA", "nCount_RNA", "percent.mt"),
                  cols = rainbow(col.num),
                  pt.size = 0.01,
                  ncol = 4) +
  theme(axis.title.x=element_blank(), axis.text.x=element_blank(),
axis.ticks.x=element_blank())
violin

scRNA.sham <- subset(scRNA.sham, subset = nFeature_RNA > 300& nFeature_RNA
< 7500 & percent.mt < 10 & nCount_RNA < 100000)

scRNA.sham <- NormalizeData(scRNA.sham)
scRNA.sham <- FindVariableFeatures(scRNA.sham, selection.method =
"vst",nfeatures = 2000)

cc.genes
CaseMatch(c(cc.genes$s.genes,cc.genes$g2m.genes),VariableFeatures(scRNA.sham))
g2m_genes = cc.genes$g2m.genes
library(Hmisc)
g2m_genes=tolower(g2m_genes)
g2m_genes=capitalize(g2m_genes)
```

```
g2m_genes = CaseMatch(search = g2m_genes, match = rownames(scRNA.sham))
s_genes = cc.genes$s.genes
s_genes=tolower(s_genes)
s_genes=capitalize(s_genes)
s_genes = CaseMatch(search = s_genes, match = rownames(scRNA.sham))
scRNA.sham<-CellCycleScoring(object=scRNA.sham,
g2m.features=g2m_genes,s.features=s_genes)

############################################
scRNA.CLP.24[["percent.mt"]] <- PercentageFeatureSet(scRNA.CLP.24, pattern =
"^mt-")
col.num <- length(levels(scRNA.CLP.24@active.ident))
violin <- VlnPlot(scRNA.CLP.24,
                  features = c("nFeature_RNA", "nCount_RNA", "percent.mt"),
                  cols = rainbow(col.num),
                  pt.size = 0.01,
                  ncol = 4) +
  theme(axis.title.x=element_blank(), axis.text.x=element_blank(),
axis.ticks.x=element_blank())
violin

scRNA.CLP.24 <- subset(scRNA.CLP.24, subset = nFeature_RNA > 300&
nFeature_RNA < 7500 & percent.mt < 10 & nCount_RNA < 100000)

scRNA.CLP.24 <- NormalizeData(scRNA.CLP.24)
scRNA.CLP.24 <- FindVariableFeatures(scRNA.CLP.24, selection.method =
"vst",nfeatures = 2000)

cc.genes
CaseMatch(c(cc.genes$s.genes,cc.genes$g2m.genes),VariableFeatures(scRNA.CLP.2
4))
g2m_genes = cc.genes$g2m.genes
library(Hmisc)
g2m_genes=tolower(g2m_genes)
g2m_genes=capitalize(g2m_genes)
g2m_genes = CaseMatch(search = g2m_genes, match = rownames(scRNA.CLP.24))
s_genes = cc.genes$s.genes
s_genes=tolower(s_genes)
s_genes=capitalize(s_genes)
s_genes = CaseMatch(search = s_genes, match = rownames(scRNA.CLP.24))
scRNA.CLP.24<-CellCycleScoring(object=scRNA.CLP.24,
g2m.features=g2m_genes,s.features=s_genes)

#################################################################
```

```
scRNA.CLP.48[["percent.mt"]] <- PercentageFeatureSet(scRNA.CLP.48, pattern = "^mt-")
col.num <- length(levels(scRNA.CLP.48@active.ident))
violin <- VlnPlot(scRNA.CLP.48,
                  features = c("nFeature_RNA", "nCount_RNA", "percent.mt"),
                  cols = rainbow(col.num),
                  pt.size = 0.01,
                  ncol = 4) +
  theme(axis.title.x=element_blank(), axis.text.x=element_blank(), axis.ticks.x=element_blank())
violin

scRNA.CLP.48 <- subset(scRNA.CLP.48, subset = nFeature_RNA > 300& nFeature_RNA < 7500 & percent.mt < 10 & nCount_RNA < 100000)

scRNA.CLP.48 <- NormalizeData(scRNA.CLP.48)
scRNA.CLP.48 <- FindVariableFeatures(scRNA.CLP.48, selection.method = "vst",nfeatures = 2000)

cc.genes
CaseMatch(c(cc.genes$s.genes,cc.genes$g2m.genes),VariableFeatures(scRNA.CLP.48))
g2m_genes = cc.genes$g2m.genes
library(Hmisc)
g2m_genes=tolower(g2m_genes)
g2m_genes=capitalize(g2m_genes)
g2m_genes = CaseMatch(search = g2m_genes, match = rownames(scRNA.CLP.48))
s_genes = cc.genes$s.genes
s_genes=tolower(s_genes)
s_genes=capitalize(s_genes)
s_genes = CaseMatch(search = s_genes, match = rownames(scRNA.CLP.48))
scRNA.CLP.48<-CellCycleScoring(object=scRNA.CLP.48, g2m.features=g2m_genes,s.features=s_genes)

##########################################################################
scRNA.sham@meta.data$orig.ident<-"sham"
scRNA.CLP.24@meta.data$orig.ident<-"CLP.24"
scRNA.CLP.48@meta.data$orig.ident<-"CLP.48"
scRNA_harmony <- merge(scRNA.sham, y=c(scRNA.CLP.24,scRNA.CLP.48))
scRNA_harmony <- NormalizeData(scRNA_harmony) %>% FindVariableFeatures() %>% ScaleData(.,vars.to.regress = c("S.Score", "G2M.Score")) %>% RunPCA(verbose=FALSE)
system.time({scRNA_harmony <- RunHarmony(scRNA_harmony, group.by.vars = "orig.ident")})
```

```
plot1 <- DimPlot(scRNA_harmony, reduction = "harmony", group.by="orig.ident")

plot2 <- ElbowPlot(scRNA_harmony, ndims=17, reduction="harmony")
save(scRNA_harmony,file = "scRNA.harmony.rdata")

scRNA_harmony <- FindNeighbors(scRNA_harmony, reduction = "harmony", dims =
1:17) %>% FindClusters(resolution = 1.2)

scRNA_harmony <- RunUMAP(scRNA_harmony, reduction = "harmony", dims =
1:17)
scRNA_harmony <- RunTSNE(scRNA_harmony, reduction = "harmony", dims = 1:17)
plot1 =DimPlot(scRNA_harmony, reduction = "tsne",label = T,split.by = "orig.ident")
plot2 = DimPlot(scRNA_harmony, reduction = "umap", label = T,split.by = "orig.ident")
#############################################################
scRNA_harmony@meta.data$celltype = "NA"
for(i in 1:nrow(celltype)){
  scRNA_harmony@meta.data[which(scRNA_harmony@meta.data$seurat_clusters
== celltype$ClusterID[i]),'celltype'] <- celltype$celltype[i]}
scRNA_harmony@meta.data$orig.ident<-
factor(scRNA_harmony@meta.data$orig.ident,levels=c('sham',"CLP.24","CLP.48"))
DimPlot(scRNA_harmony, reduction = "umap", group.by = "celltype",label =
T,split.by = 'orig.ident')
```

```
Sys.setenv(LANGUAGE = "en")
options(stringsAsFactors = FALSE)
rm(list=ls())

library(Seurat)
library(tidyverse)
library(dplyr)
library(patchwork)
library(harmony)
load("CD45-.rdata")
Idents(CD45_)<-"celltype"
Endothelial<-subset(CD45_,idents = "Endothelial cells")
plot1 <- DimPlot(Endothelial, reduction = "pca", group.by="orig.ident")

plot2 <- ElbowPlot(Endothelial, ndims=15, reduction="pca")

table(Endothelial@meta.data$seurat_clusters)

Endothelial <- FindNeighbors(Endothelial, reduction = "pca", dims = 1:12) %>%
FindClusters(resolution = 0.25)
Endothelial <- RunTSNE(Endothelial, reduction = "pca", dims = 1:12)
Endothelial <- RunUMAP(Endothelial, reduction = "pca", dims = 1:12)
?DimPlot
DimPlot(Endothelial, reduction = "tsne",label = T)
DimPlot(Endothelial, reduction = "umap", label = T)
save(Endothelial,file = "Endothelial.rdata")
################################################
celltype = data.frame(ClusterID=c(0,1,2,3,4,5,6),
celltype=c("Capillary","Unkown","Capillary","Aerocyte","Lymphatic","Vein","Artery
"), stringsAsFactors = FALSE)
Endothelial@meta.data$celltype = "NA"
for(i in 1:nrow(celltype)){
  Endothelial@meta.data[which(Endothelial@meta.data$seurat_clusters ==
celltype$ClusterID[i]),'celltype'] <- celltype$celltype[i]}

x=Endothelial@meta.data$celltype
library(data.table)
table(x)
pB2_df <-
table(Endothelial@meta.data$celltype,Endothelial@meta.data$orig.ident) %>% melt()
####################################
FeaturePlot(Endothelial,features = "Fxyd6",reduction = "umap",pt.size = 0.5)+
  theme_bw()+
  theme()
```

```
FeaturePlot(Endothelial,features = "Mmrn1",reduction = "umap",pt.size = 0.5)+
   theme_bw()+
   theme()
FeaturePlot(Endothelial,features = "Car4",reduction = "umap",pt.size = 0.5)+
   theme_bw()+
   theme()
FeaturePlot(Endothelial,features = "Lpl",reduction = "umap",pt.size = 0.5)+
   theme_bw()+
   theme()
FeaturePlot(Endothelial,features = "Fibin",reduction = "umap",pt.size = 0.5)+
   theme_bw()+
   theme()
FeaturePlot(Endothelial,features = "Slc6a2",reduction = "umap",pt.size = 0.5)+
   theme_bw()+
   theme()
FeaturePlot(Endothelial,features = "Bst1",reduction = "umap",pt.size = 0.5)+
   theme_bw()+
   theme()
FeaturePlot(Endothelial,features = "Mgp",reduction = "umap",pt.size = 0.5)+
   theme_bw()+
   theme()
```

```
Sys.setenv(LANGUAGE = "en")
options(stringsAsFactors = FALSE)
rm(list=ls())

setwd("F:/ GSE207651_RAW")
library(Seurat)
library(tidyverse)
library(dplyr)
library(patchwork)
library(devtools)
library(harmony)
load("scRNA.harmony.rdata")
CD45=subset(scRNA.harmony,idents =
c("Granulocytes","Monocytes","Macrophages","NK cells","DC cells","T cells","B
cells"))
CD45_ =subset(scRNA.harmony,idents = c("Fibroblasts","Endothelial cells","Smooth
muscle cells","Epithelial cells"))
##################################
x=CD45@meta.data$celltype
library(data.table)
table(x)
pB2_df <- table(CD45@meta.data$celltype,CD45@meta.data$orig.ident) %>% melt()
colnames(pB2_df) <- c("Cluster","Sample","Number")
cluster=c("Granulocytes","Monocytes","Macrophages","NK cells","DC cells","T
cells","B cells")
pB2_df$Cluster <- factor(pB2_df$Cluster,levels = cluster)
sample_color <-
c("#d95f02","#66a61e","#1b9e77","#e7298a","#386cb0","red3","#8dd3c7")

Idents(CD45)<-"orig.ident"
CD45@meta.data$orig.ident<-factor(CD45@meta.data$orig.ident,levels =
c("CLP.48","CLP.24","sham"))

pB4 <- ggplot(data = pB2_df, aes(x =Number, y = Sample, fill =  Cluster)) +
  geom_bar(stat = "identity", width=0.8,position="fill")+
  scale_fill_manual(values=sample_color) +
  theme_bw()+
  theme(panel.grid =element_blank()) +
  labs(x="",y="Ratio")+
  theme(axis.text.y = element_text(size=12, colour = "black"))+
  theme(axis.text.x = element_text(size=12, colour = "black"))
pB4
##########################################
x=CD45_@meta.data$celltype
```

```
library(data.table)
table(x)
pB2_df <- table(CD45_@meta.data$celltype,CD45_@meta.data$orig.ident) %>%
melt()
colnames(pB2_df) <- c("Cluster","Sample","Number")
cluster= c("Fibroblasts","Endothelial cells","Smooth muscle cells","Epithelial cells")
pB2_df$Cluster <- factor(pB2_df$Cluster,levels = cluster)
sample_color <-
c("#d95f02","#66a61e","#1b9e77","#e7298a","#386cb0","red3","#8dd3c7")
Idents(CD45_)<-"orig.ident"
CD45_@meta.data$orig.ident<-factor(CD45_@meta.data$orig.ident,levels =
c("CLP.48","CLP.24","sham"))

pB4 <- ggplot(data = pB2_df, aes(x =Number, y = Sample, fill =  Cluster)) +
  geom_bar(stat = "identity", width=0.8,position="fill")+
  scale_fill_manual(values=sample_color) +
  theme_bw()+
  theme(panel.grid =element_blank()) +
  labs(x="",y="Ratio")+
  theme(axis.text.y = element_text(size=12, colour = "black"))+
  theme(axis.text.x = element_text(size=12, colour = "black"))
pB4
#####################################################
library(data.table)
counts=fread("细胞数量改变.csv",data.table = F)


rownames(counts)=counts$V1
counts=counts[-4,-1]
counts=as.matrix(counts)
barplot(counts,legend=rownames(counts),beside = T,
        ylab = "Number of cells",col = c("#FFFFCC","#CCFFFF","#FFCCCC"))
barplot(counts,legend=rownames(counts),beside = T,
        ylab = "Number of cells",col = c("#FFFFFF","#CCCCCC","66699"))
barplot(counts,legend=rownames(counts),beside = T,
        ylab = "Number of cells",col = c("red","yellow","blue"))
```

```
Sys.setenv(LANGUAGE = "en")
options(stringsAsFactors = FALSE)
rm(list=ls())

library(Seurat)
library(tidyverse)
library(dplyr)
library(patchwork)
library(harmony)
library(cowplot)
load("CD45.rdata")
load("CD45-.rdata")

CD45.totalscale<-CD45
scale.genes <-    rownames(CD45.totalscale)

CD45.totalscale<-ScaleData(CD45.totalscale,features = scale.genes, vars.to.regress = c("S.Score", "G2M.Score"))

load("CD45+totalscale.rdata")
Idents(CD45.totalscale)="celltype"
CD45.totalscale@meta.data$celltype=factor(CD45.totalscale@meta.data$celltype,levels = c("B cells","T cells","NK cells","DC cells",

"Macrophages","Monocytes","Granulocytes"))

DoHeatmap(subset(CD45.totalscale,downsample=100), features = gene,label = F,slot = "scale.data")
gene=c("Cd19","Cd79a",   "Cd79b","Trbc2","Trbc1",   "Gzma","Nkg7",      "Ccl17",
"Flt3","Mrc1","Marco","Cd44","Ear2", "Plac8", "Ccr2", "F13a1",
          "S100a8","Retnlg")

load("CD45-.rdata")
Idents(CD45_)="celltype"
gene=c("Sftpa1","Ager","Cbr2", "Lamp3","Cxcl15", "Cdh5",      "Cldn5",
"Lyve1","Calcrl",
        "Tagln","Myh11","Tpm2",       "Acta2","Mustn1","Col1a2","Dcn",       "Gsn",
"Mfap4")
DoHeatmap(subset(CD45_,downsample=100), features = gene,label = F,slot = "scale.data")
```

```
Sys.setenv(LANGUAGE = "en")
options(stringsAsFactors = FALSE)
rm(list=ls())
library(ggplot2)
y=read.csv("cluster3-BP.txt",header = T,sep = "\t")
colnames(y)

mytheme<-theme(axis.text.y = element_text(size=14),axis.text.x = element_text(size = 12),
               panel.background = element_rect(fill = "white",color = "black"),
               panel.grid.major = element_line(color = "#CCCCCC",linetype = 1),
               panel.grid.minor = element_line(color = "#CCCCCC",linetype = 1))

ggplot(y,aes(x = Rich.factor,
             y = reorder(Description,Rich.factor,sum),
             size = Count,
             colour=P.value)) +
  geom_point(shape = 16) +
  labs(x = "Rich factor", y = "Pathway")+
  scale_colour_continuous(
    name="P.value",
    low="red",
    high="green")+
  scale_radius(
    range=c(3,6),
    name="Count")+
  guides(
    color = guide_colorbar(order = 1),
    size = guide_legend(order = 2)
  )+mytheme

##############################
load("CD45-.rdata")
Idents(CD45_)<-"celltype"
Endothelial<-subset(CD45_,idents = "Endothelial cells")
VlnPlot(Endothelial, features = c("Inhbb","Plat","Npr3","Spry4","Clec1a","Atp8a1","Lpin2","Arap2","Cd74","H2-Ab1","Gbp4","Cyp1a1"), pt.size = 0, ncol = 1)+
  scale_x_discrete("")+
  theme(
    axis.text.x.bottom = element_blank()
  )
##########################################################
Sys.setenv(LANGUAGE = "en")
```

```
options(stringsAsFactors = FALSE)
rm(list=ls())
library(Seurat)
load("Endothelial.rdata")
Idents(Cap)="celltype"
table(Endothelial@meta.data$celltype)
Cap=subset(Endothelial,idents =c("Capillary-1","Capillary-2","Capillary-3") )
Cap@meta.data$celltype<-as.factor(Cap@meta.data$celltype)
Cap@meta.data$celltype=factor(Cap@meta.data$celltype,levels = c("Capillary-
1","Capillary-2","Capillary-3"))
DotPlot(Cap,features = c("Cd274","Stat1","Gbp2","Ifi47","Gbp6","Igtp",
                                   "Gbp7","Gbp4","H2-T23","H2-Aa","H2-Eb1","H2-
Ab1","Cd74"))+coord_flip()+
  theme_bw()+
  theme(panel.grid = element_blank(),
        axis.text.x=element_text(angle=45,hjust = 0.5,vjust=0.5,face = "bold",size =
12),
        axis.text.y = element_text(face = "bold",size = 12))+
  labs(x=NULL,y=NULL)

DotPlot(Cap,features = c("Spry4","Igfbp4","Adamts9","Flt1","Plaur","Plat",

"Npr3","Ace","Lpin2","Nrip1","Cd36","Lpl"))+coord_flip()+
  theme_bw()+
  theme(panel.grid = element_blank(),
        axis.text.x=element_text(angle=45,hjust = 0.5,vjust=0.5,face = "bold",size =
12),
        axis.text.y = element_text(face = "bold",size = 12))+
  labs(x=NULL,y=NULL)
```

```
Sys.setenv(LANGUAGE = "en")
options(stringsAsFactors = FALSE)
rm(list=ls())

library(GSEABase)
library(clusterProfiler)
library(GSEA)
library(GSVA)
library(Seurat)
load("Endothelial.rdata")

Idents(Endothelial)<-"celltype"
Endothelial@meta.data$orig.ident<-
factor(Endothelial@meta.data$orig.ident,levels=c("sham","CLP.24","CLP.48"))
x=AverageExpression(Endothelial,add.ident = "orig.ident")

exp=x[["RNA"]]
exp1=as.matrix(exp)
library(msigdbr)
m_df<- msigdbr(species = "Mus musculus",  category = "C2", subcategory =
"KEGG")
fgsea_sets<- m_df %>% split(x = .$gene_symbol, f = .$gs_name)

es.max <- gsva(exp1, fgsea_sets, mx.diff=FALSE,kcdf="Poisson")

pheatmap::pheatmap(es.max,
                   cluster_rows = T,
                   cluster_cols =F,
                   show_colnames=T,
                   scale = "row",
                   color                 =colorRampPalette(c("#FF7744",
"white","#AAAAAA","#0044BB"))(100))
```

```
Sys.setenv(LANGUAGE = "en")
options(stringsAsFactors = FALSE)
rm(list=ls())

library(Seurat)
library(tidyverse)
library(dplyr)
library(patchwork)
library(harmony)
load("CD45-.rdata")


Idents(CD45_)="celltype"
Endothelial<-subset(CD45_,idents="Endothelial cells")
Idents(Endothelial)<-"orig.ident"
deg=FindMarkers(Endothelial,ident.1 = "CLP.24",ident.2 = "sham",
                min.pct = 0.01,logfc.threshold = 0.01)

genelist=deg$avg_log2FC
names(genelist)=toupper(rownames(deg))

genelist=sort(genelist,decreasing = T)
library(ggplot2)
library(clusterProfiler)
library(org.Hs.eg.db)

library(enrichplot)
geneset=read.gmt("c2.cp.kegg.v7.5.1.symbols.gmt")
length(unique(geneset$term))
egmt=GSEA(genelist,TERM2GENE = geneset,
          minGSSize = 1,pvalueCutoff = 0.5)

gseaplot2(egmt,geneSetID = 1,pvalue_table = T)


x=egmt@result
write.csv(x,file = "GSEA-Endothelial.csv")
##########################################################################
library(msigdbr)
b=str_split(x$ID,pattern = "KEGG_",simplify = T)[,2]
x$ID<-b

p=ggplot(x %>% filter(p.adjust < 0.05) %>% head(n= 21), aes(reorder(ID, NES), NES))
+
```

```
    geom_point(aes(size=setSize,color=p.adjust)) +
    coord_flip() +
    labs(x="Pathway", y="Normalized Enrichment Score",
            title="Hallmark pathways NES from GSEA") +
    theme_minimal()
p
p+scale_color_continuous(low='red' ,high='blue')+theme_bw()
```

```
Sys.setenv(LANGUAGE = "en")
options(stringsAsFactors = FALSE)
rm(list=ls())

library(Seurat)
library(tidyverse)
library(dplyr)
library(patchwork)
library(harmony)
library(cowplot)
load("CD45+.rdata")
Idents(CD45)<-CD45@meta.data$celltype
c1 <- subset(CD45,idents = "B cells")
Idents(c1) <- "orig.ident"
table(c1@meta.data$orig.ident)


c1.deg=FindMarkers(c1,ident.1 = "CLP.48",ident.2 = "CLP.24")
log2FC<-abs(c1.deg$avg_log2FC)>1.2
adj_p<-c1.deg$p_val_adj<0.05
c1.deg.1<-c1.deg[log2FC&adj_p,]
library(ggplot2)
library(ggrepel)

avg.c1 <- log1p(AverageExpression(c1, verbose = FALSE)$RNA)
avg.c1<-as.data.frame(avg.c1)
avg.c1$gene <- rownames(avg.c1)
genes.to.label = rownames(c1.deg.1)


avg.c1$Sig="NO_DIFF"
avg.c1$Sig[avg.c1$gene %in%genes.to.label]="DIFF"

p=ggplot(avg.c1, aes(sham, CLP.24))+xlim(0,4.5)+ylim(0,4.5)+
  geom_point(aes(color=Sig),size=1)+theme_classic()+
  scale_color_manual(values = c("#f53f38","#b6bbc2"))
data_selected <- avg.c1[rownames(c1.deg.1),]
p + geom_label_repel(data=data_selected,label.size=0.1,size =3,
                     aes(label=rownames(data_selected)),max.overlaps =
20)+ggtitle("Endothelial cells") + theme(plot.title = element_text(hjust = 0.5))
```

```
Sys.setenv(LANGUAGE = "en")
options(stringsAsFactors = FALSE)
rm(list=ls())
library(CellChat)
library(tidyverse)
library(ggalluvial)
library(Seurat)
library(data.table)
library(ggsci)

load("T cells.rdata")
load("Endothelial.rdata")
colnames(scRNA_harmony@meta.data)[13]<-"celltype1"
Endothelial@meta.data$celltype<-
paste("cluster",Endothelial@meta.data$seurat_clusters,sep = "-")

Endo_Tcell<-merge(Endothelial,y=Tcell)

data.input <- GetAssayData(Endo_Tcell, assay = "RNA", slot = "data")
identity <- subset(Endo_Tcell@meta.data, select = "celltype")
cellchat <- createCellChat(object = data.input, meta = identity,    group.by = "celltype")

sc.sp=SplitObject(Endo_Tcell,split.by = "orig.ident")
sc.sham=sc.sp[[1]]
sc.CLP.24=sc.sp[[2]]
sc.CLP.48=sc.sp[[3]]

table(sc.CLP.48@meta.data$celltype)
which(cc.sham@meta$celltype=="cluster-0")
cc.sham@meta<-cc.sham@meta[-1046,]

sc.sham@meta.data$celltype[sc.sham@meta.data$celltype=="cluster-0"]<-"cluster-
3/0"
sc.CLP.48@meta.data$celltype[sc.CLP.48@meta.data$celltype=="cluster-3"]<-
"cluster-3/0"
sc.CLP.24@meta.data$celltype[sc.CLP.24@meta.data$celltype=="cluster-3"]<-
"cluster-3/0"

cellchat.48<- createCellChat(object =sc.CLP.48@assays$RNA@data, meta
=sc.CLP.48@meta.data,    group.by ="celltype")
dir.create("compare-2")
setwd("compare-2/")
CellChatDB <- CellChatDB.mouse
cellchat=cellchat.48
```

```
cellchat@DB    <- CellChatDB
cellchat <- subsetData(cellchat)
future::plan("multiprocess", workers = 4)
cellchat <- identifyOverExpressedGenes(cellchat)
cellchat <- identifyOverExpressedInteractions(cellchat)
cellchat <- computeCommunProb(cellchat, raw.use = TRUE,population.size =T)
cellchat <- filterCommunication(cellchat, min.cells = 3)
df.net<-subsetCommunication(cellchat)
write.csv(df.net,file = "Endo-Tcell-48_signal_net.csv")

cellchat <- computeCommunProbPathway(cellchat)
df.netp<-subsetCommunication(cellchat,slot.name = "netP")
write.csv(df.netp,file = "Endo-Tcell-48_signal_netp.csv")

cellchat <- aggregateNet(cellchat)
cellchat <- netAnalysis_computeCentrality(cellchat, slot.name = "netP")

cc.48 = cellchat
save(cc.48,file = "Endo-Tcell-cellchat_48_singal.rdata")


cc.list=list(sham=cc.sham,CLP.24=cc.24)
cellchat=mergeCellChat(cc.list,cell.prefix = T,add.names = names(cc.list))
save(cellchat,file = "cellchat.shamvs24.rdata")

gg1<-compareInteractions(cellchat,show.legend = F,group = c(1,2),measure = "count")
gg2<-compareInteractions(cellchat,show.legend = F,group = c(1,2),measure = "weight")
gg1+gg2

par(mfrow = c(1,2), xpd=TRUE)
netVisual_diffInteraction(cellchat,weight.scale = T)
netVisual_diffInteraction(cellchat,weight.scale = T,measure = "weight")


weight.max=getMaxWeight(cc.list,attribute = c("idents","count"))
netVisual_circle(cc.list[[1]]@net$count,weight.scale = T,label.edge = F,
                         edge.weight.max         =weight.max[2],edge.width.max          =
12,title.name = "sham" )

netVisual_circle(cc.list[[2]]@net$count,weight.scale = T,label.edge = F,
                         edge.weight.max         =weight.max[2],edge.width.max          =
12,title.name = "CLP" )
```

```
unique(cellchat@meta$celltype)
group.cellType <- c("cluster-2", "cluster-3/0", "T cells","cluster-1")
group.cellType <- factor(group.cellType, levels = c("cluster-3/0", "cluster-2","T cells","cluster-1"))
cc.list <- lapply(cc.list, function(x) {mergeInteractions(x, group.cellType)})
cellchat <- mergeCellChat(cc.list, add.names = names(cc.list))

par(mfrow = c(1,2), xpd=TRUE)
netVisual_diffInteraction(cellchat, weight.scale = T, measure = "count.merged", label.edge = T)
netVisual_diffInteraction(cellchat, weight.scale = T, measure = "weight.merged", label.edge = T)

netVisual_bubble(cellchat, sources.use = c(2:3), targets.use = 7,    comparison = c(1, 2), angle.x = 45)
```

```
library(AUCell)
library(ggplot2)
library(Seurat)
library(clusterProfiler)
library(msigdbr)

cells_rankings <- AUCell_buildRankings(Endothelial@assays$RNA@data,
nCores=6, plotStats=TRUE)

cells_rankings

c2 <- read.gmt("c2.cp.kegg.v7.5.1.symbols.gmt")
geneSets <- lapply(unique(c2$term), function(x){print(x);c2$gene[c2$term == x]})
names(geneSets) <- unique(c2$term)

m_df<- msigdbr(species = "Mus musculus",  category = "C2", subcategory =
"KEGG")
fgsea_sets<- m_df %>% split(x = .$gene_symbol, f = .$gs_name)


cells_AUC <- AUCell_calcAUC(fgsea_sets, cells_rankings,nCores =1,
aucMaxRank=nrow(cells_rankings)*0.1)
grep("COMPLEMENT",rownames(cells_AUC@assays@data$AUC),value = T)

geneSet <- "KEGG_COMPLEMENT_AND_COAGULATION_CASCADES"
aucs <- as.numeric(getAUC(cells_AUC)[geneSet, ])
Endothelial$AUC <- aucs
df<- data.frame(Endothelial@meta.data,
Endothelial@reductions$umap@cell.embeddings)
colnames(df)
class_avg <- df %>%
  group_by( seurat_clusters) %>%
  summarise(
    UMAP_1 = median(UMAP_1),
    UMAP_2 = median(UMAP_2)
  )

ggplot(df, aes(UMAP_1, UMAP_2))  +
  geom_point(aes(colour  = AUC)) + viridis::scale_color_viridis(option="A") +
  ggrepel::geom_label_repel(aes(label = seurat_clusters),
                            data = class_avg,
                            size = 5,
                            label.size = 1,
                            segment.color = NA
```

```
)+      theme(legend.position = "none") + theme_bw()
```
