# Supplementary figures and images for: Lung single-cell RNA profiling reveals response of pulmonary capillary to sepsis-induced acute lung injury
Source: Front Immunol. 2024 Jan 29;15:1308915. doi: 10.3389/fimmu.2024.1308915 (PMC10859485; doi:10.3389/fimmu.2024.1308915)

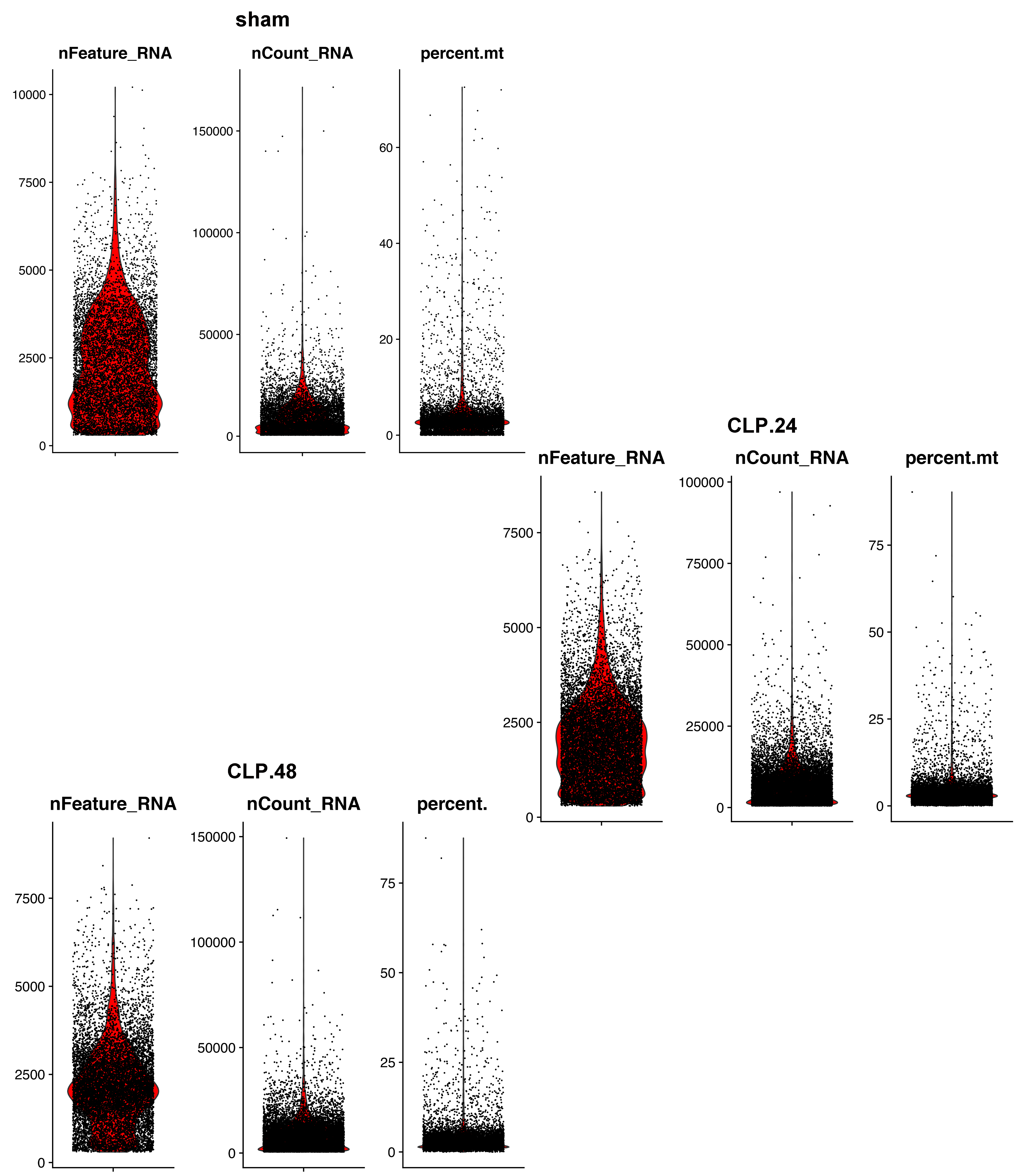

Supplement: Supplementary file 2 [file Image_1.tif]
